# Oral and Gastric Helicobacter Pylori: Effects and Associations

**DOI:** 10.1371/journal.pone.0126923

**Published:** 2015-05-26

**Authors:** Nélio Veiga, Carlos Pereira, Carlos Resende, Odete Amaral, Manuela Ferreira, Paula Nelas, Claudia Chaves, João Duarte, Luis Cirnes, José Carlos Machado, Paula Ferreira, Ilídio J. Correia

**Affiliations:** 1 Health Sciences Research Centre, Health Sciences Faculty, Beira Interior University, Covilhã, Portugal; 2 Research Centre for Education, Technology and Health Studies, Polytechnic Institute of Viseu, Viseu, Portugal; 3 Chemical Process Engineering and Forest Products Research Centre, Chemical Engineering Department, University of Coimbra, Coimbra, Portugal; 4 Health Sciences Department, Portuguese Catholic University, Viseu, Portugal; 5 Institute of Molecular Pathology and Immunology of the University of Porto, Porto, Portugal; Second University of Naples, ITALY

## Abstract

**Introduction:**

This study consisted in the comparison of the prevalence of *Helicobacter pylori (H*. *pylori)* present in the stomach and in saliva of a sample of Portuguese adolescents and the assessment of the association between *H*. *pylori* infection with socio-demographic variables and prevalence of dental caries.

**Materials and Methods:**

A cross-sectional study was designed including a sample of 447 adolescents aged 12 to 19 years old, attending a public school in Sátão, Portugal. A questionnaire about socio-demographic variables and oral health behaviors was applied. Gastric *H*. *pylori* infection was determined using the urease breath test (UBT). Saliva collection was obtained and DNA was extracted by Polymerase Chain Reaction (PCR) in order to detect the presence of oral *H*. *pylori*.

**Results:**

The prevalence of gastric *H*. *pylori* detected by UBT was 35.9%. Within the adolescents with a gastric UBT positive, only 1.9% were positive for oral *H*. *pylori*. The presence of gastric *H*. *pylori* was found to be associated with age (>15years, *Odds ratio *(OR)=1.64,95%CI=1.08-2.52), residence area (urban,OR=1.48,95%CI=1.03-2.29) and parents´ professional situation (unemployed,OR=1.22,95%CI=1.02-1.23). Among those with detected dental caries during the intra-oral observation, 37.4% were positive for gastric *H*. *pylori* and 40.2% negative for the same bacterial strain (p=0.3).

**Conclusions:**

The oral cavity cannot be considered a *reservoir* for infection of *H*. *pylori*. Gastric *H*. *pylori* infection was found to be associated with socio-demographic variables such as age, residence area and socioeconomic status.

## Introduction

An important bacterium species that may have an important pathobiological role, especially in the gastric mucosa, is *Helicobacter pylori* (*H*. *pylori*)—a gram-negative, microaerophilic, rod-shaped bacterium that colonizes the human stomach.[1,2] It resides beneath the gastric mucous layer, adjacent to the gastric epithelial cells, and, although it is not invasive, it can give rise to chronic inflammation of the gastric mucosa.[3] Infection with this organism is now recognized as a serious, transmissible infectious disease, highly associated with the development of chronic and atrophic gastritis, duodenal ulcers, gastric mucosa-associated lymphoid tissue (MALT) lymphoma, and gastric carcinoma.[4] The recognition of the pathological aspects of *H*. *pylori* infection in the upper gastro-intestinal tract, was firstly originated from a series of studies performed by Marshal and Warren in 1982.[3]

Various studies demonstrate that approximately 50% of the world’s population may be infected, with developed countries showing a lower prevalence of *H*. *pylori* infection at all ages when compared with developing countries. Notably, the difference in the infection pattern is especially noticeable among younger people. The fecal-oral tract is believed to be a common route of transmission of *H*. *pylori*, so demographic areas with reduced levels of sanitation and low socioeconomic status are associated with an increased prevalence of *H*. *pylori* infection.[5,6]

A study developed in 2012 by Almeida *et al*. in Portugal, revealed a prevalence of 40.6% of *H*. *pylori* infection among portuguese schoolchildren between 11 and 18 years old, but with a tendency to decrease in the following years.[7] Relevantly, the worldwide infection by *H*. *pylori* has been decreasing due to better sanitary and socioeconomic conditions, and the same should happen in Portugal.

It has long been speculated that dental plaque might harbour *H*. *pylori*, and, by that reason, it can be a source of re-infection of the gastric mucosa. Additionally, the presence of the bacteria in the oral cavity was suggested to be associated with a higher risk of dental caries development.[8–10] Some studies have also shown that the presence of *H*. *pylori* in the periodontium may be one of the main causes of periodontal infection.[11–14] Other studies demonstrate the association between inadequate oral hygiene habits and the presence of oral *H*. *pylori* in the dental plaque.[13–16] Further, some studies indicate that the presence of *H*. *pylori* in the oral cavity can be associated with gastro-esophageal infection, suggesting the mouth as the first extra-gastric *reservoir* for *H*. *pylori*.[12,17] Therefore, the failure to eliminate *H*. *pylori* present in the oral cavity can lead to gastrointestinal re-infection.[17]

Moreover, other authors suggest that *H*. *pylori* may belong to the normal oral flora of the human oral cavity, maintaining a commensal relation with the host, but present in very low numbers such that reliable identification is difficult.[18,19] The difficulty in establishing a connection between the role of *H*. *pylori* in the oral cavity and gastric infection remains controversial, since the detection rate of the bacterium in the mouth is very diverse, ranging between 0% and 100%.[4]

The two main aims of our work were: (i) to explore the hypothesis if the oral cavity may be considered a potential *reservoir* for *H*. *pylori*—to assess this we performed the detection of gastric *H*. *pylori* through Urease Breath Test (UBT) followed by the detection of oral *H*. *pylori* among those with positive UBT by Polymerase Chain Reaction (PCR) in a sample of Portuguese adolescents; (ii) to determine the association between *H*. *pylori* infection and socio-demographic variables, and the prevalence of dental caries.

## Materials and Methods

### Material collection

A non-probabilistic convenience sample of 447 adolescents aged between 12 and 19 years old, attending a public school in Sátão, Portugal, was enrolled in this study. All samples were obtained from September to December of 2012. Questionnaires without information about gender and age were excluded of the study as well as the adolescents whose parents did not sign the informed consent before data collection.

A self-administered questionnaire focusing socio-demographic variables, social and daily habits and oral health behaviors was filled out by all participants in this study. Questions about socio-demographic variables such as gender (male/female), age, school grade at the moment of the study, residence area (urban/rural), parents´ educational level (choosing the higher educational level between father and mother), parents´ professional situation (employed/unemployed) and the number of rooms and people living in the house were used to determine the crowding index.

This research has been performed in accordance with the Declaration of Helsinki and was submitted and approved by the Ethics Committee of the Health School and Research Centre for Education, Technology and Health Studies of the Polytechnic Institute of Viseu, Portugal (CI&DETS). The information collected by the questionnaires was provided voluntarily and confidentially, guaranteeing, anonymity of the information collected by telling the adolescents not to sign their names or write down any other form of identification in any part of the questionnaire. Data collection was only made on adolescents from whom we obtained written informed consent from the next of kin, caretakers, or guardians on behalf of the minors that were enrolled in the present study. After collection, the questionnaires were numbered, stored and processed by computer. The results do not refer to nominal adolescents or contain any information that may identify any of the participants.

### Clinical sample characterization

Clinical examination of oral health status was carried out according to the World Health Organization (WHO) criteria.[20] The teeth were clinically examined with dental instruments using visual-tactile methods with the use of a dental mirror and a probe (approved by the WHO for caries diagnosis) and took place in the classroom under standardized conditions recommended by the WHO. Cotton rolls and gauze were available to remove moisture and plaque when necessary. There was only one observer that registered the results of each clinical observation during the study. The recorded variables of the clinical examination were caries experience, using the decayed, missing and filled permanent tooth index (DMFT) as an oral health indicator, which consists in the sum of teeth decayed, teeth missing due to dental caries and teeth filled for each analyzed adolescent. Each tooth would be classified with only one of the following codes: 0—sound crown or root, showing no evidence of either treated or untreated caries; 1—indicates a tooth with caries; 2—filled teeth, with additional decay; 3—filled tooth with no decay; 4—tooth that is missing as a result of caries; 5—a permanent tooth missing for any other reason than decay; 6—teeth on which sealants have been placed; 7—indicate that the tooth is part of a fixed bridge; 8—this code is used for a space with an unerupted permanent tooth where no primary tooth is present; 9—erupted teeth that cannot be examined; T—indicates trauma in the presence of a fractured crown.

### *H*. *pylori* detection

From the total sample of 447 adolescents, 437 were screened for gastric *H*. *pylori* infection using the UBT that consists in the exhalation of carbon dioxide in samples before and after swallowing urea labeled with non-radioactive carbon-13. The samples were then analyzed and each result would be classified as positive or negative for *H*. *pylori* infection.

To detect *H*. *pylori* on oral cavity, saliva was collected by the passive drool method into a polypropylene tube until reaching 2 milliliters of saliva in each tube per adolescent. Next, DNA was extracted using the MagNA Pure LC DNA Isolation Kit (Bacteria, Fungi) (Roche), quantified with Nanodrop (Thermo Lifesciences), and bacterial DNA was amplified using the Multiplex PCR kit (Qiagen) with primers that recognize all *H*.*pylori* strains: VacA_Fw: ATGGAAATACAACAAACACAC and VacA_Rv: CTGCTTGAATGCGCCAAAC(21). PCR products were observed, after electrophoresis in a 2% agarose gel stained with RedSafe (Intron), in a UV chamber (Bio-Rad). DNA bands with the expected molecular weight were excised from the agarose gel, purified with ULTRAPrep Agarose Gel Extraction kits (AHN), and sequenced with BigDye Terminator Sequencing Kit (Applied Biosystems). The obtained DNA sequences were compared with a control (*H*. *pylori* positive DNA sample diluted in saliva at different concentrations) and with NCBI database (http://www.ncbi.nlm.nih.gov/).

### Statistical analysis

Data analysis was carried out using the *Statistical Package for Social Sciences* (SPSS 18.0 version). Prevalence was expressed in proportions and crude odds ratio (OR) with 95% confidence intervals (CI) were used to measure the strength of association between variables. Proportions were compared by the Chi-square test. The significance level established the inferential statistics was 5% (p<0.05).

## Results

The sample used in this study was composed by 447 adolescents, 38.3% male gender and 61.7% female gender, with ages between 12 and 19 years old, from a public school of Sátão, Portugal. When analyzing the parents’ educational level, we could verify that 4.3% of participants have parents that only frequented school for less than the 4^th^ grade, 53.5% stayed in school from the 5^th^ to the 12^th^ grade and 15.0% went to a superior degree after finishing the 12^th^ grade. Crowding index < 1.0 is presented among 71.4% of adolescents, while 14.1% are equal to 1 and only 4.5% > 1.0, which indicates possible overcrowding at home. Performing the analysis of the distribution of the sample by residential area, we could observe that the majority of participants live in rural areas (65.3% vs 34.7%) (Table 1).

**Table 1:** Sample characterization.

	Male		Female		Total	
	N	%	N	%	N	%
	171	38.3	276	61.7	447	100,0
Age						
12	31	18.2	27	9.8	58	13.0
13	29	17.0	38	13.8	67	15.0
14	18	10.5	34	12.3	52	11.6
15	28	16.4	27	9.8	55	12.3
16	16	9.4	34	12.3	50	112
17	16	9.4	29	10.5	45	10.1
≥18	33	12.3	87	24.3	120	26.6
Grade						
7	34	19.9	26	9.4	59	13.4
8	26	15.2	38	13.8	64	14.3
9	19	11.1	33	12.0	52	11.6
10	30	17.5	38	13.8	68	15.2
11	19	11.1	32	11.6	51	11.4
12	44	25.2	109	39.4	153	34.1
Parents´educational level						
≤ 4 grade	3	2.0	16	6.9	19	4.3
5–12 grade	105	61.4	134	48.6	239	53.5
> 12 grade	30	17.5	37	13.4	67	15.0
Without information	33	19.1	89	31.1	122	27.2
Crowding índex						
<1.0	110	64.3	209	75.7	319	71.4
1,0	25	14.6	38	13.8	63	14.1
>1.0	8	4.7	12	4.3	20	4.5
Without information	28	16.4	17	6.2	45	10.0
Residential area						
Rural	100	58.5	192	69.6	292	65.3
Urban	71	41.5	84	30.4	155	34.7

Furthermore, the prevalence of gastric *H*. *pylori* detected by the UBT was 35.9% (157 positive individuals from a total of 437) (Table A in S1 File).

The presence of gastric *H*. *pylori* was associated with age, residence area and parents´ professional situation, registering a higher prevalence among older adolescents (OR = 1.64, 95%CI = 1.08–2.52), those who live in urban areas (OR = 1.48, 95%CI = 1.03–2.29) and among the adolescents whose parents are unemployed at the moment of data collection (OR = 1.22, 95%CI = 1.02–1.23) (Table 2).

**Table 2:** Association between the presence of gastric H. pylori (detected = positive test; non-detected = negative test) and socio-demographic variables.

	Positive test		Negative test		
	N	%	N	%	*p*
**Gender**					
Male	57	34.8	107	65.2	
Female	100	36.6	173	63.4	0.4
**Age**					
≤ 15 years	72	34.1	139	65.9	
> 15 years	67	45.9	79	54.1	**0.02**
**Residential area**					
Rural	79	35.4	144	64.6	
Urban	60	44.8	74	55.2	**0.04**
**Parents´educational level**					
≤9^th^ grade	90	38.5	144	61.5	
>9^th^ grade	53	36.1	94	63.9	0.4
**Parents´professional situation**					
Employed	89	36.6	154	63.4	
Unemployed	43	41.3	61	58.7	**0.04**

There is also no correlation between the presence of gastric *H*. *pylori* and the prevalence of dental caries. The results of the present study demonstrated that, among caries-free adolescents, 62.6% were positive for gastric *H*. *pylori* and 59.8% were negative (Table B in S1 File). Among those with detected dental caries during the intra-oral observation, 37.4% were positive for gastric *H*. *pylori* and 40.2% negative for the same bacteria in study (p = 0.3).

**Fig 1:**
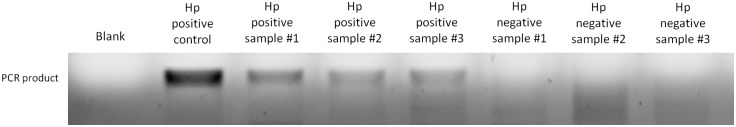
*H*. *pylori*- specific PCR for DNA extracted from oral cavity. In this figure, one can see the amplification of *VacA* in the three cases previously identified as oral cavity positives for H. pylori (Hp positive sample #1 to #3), in comparison with other three cases that are oral cavity H. pylori negatives (Hp negative sample #1 to #3). As a positive control (Hp positive control) PCR for DNA extracted from *H*. *pylori* (strain 7354) diluted in saliva was used. Blank—PCR negative control.

In order to detect the presence of *H*. *pylori* in the oral cavity, we performed PCR of the DNA extracted from saliva of positive UBT participants. Interestingly, from the individuals with positive UBT, indicating gastric *H*. *pylori* infection, only 1.9% were positive for oral *H*. *pylori* (Fig 1) (Table C in S1 File).

**Fig 2:**
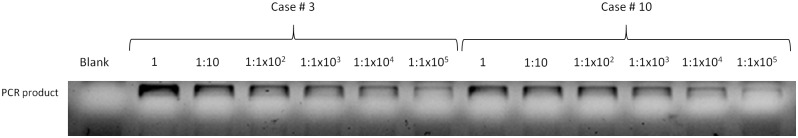
Highly sensitive PCR for detection of *H*. *pylori*. In order to evaluate the sensitivity of *VacA*-specific PCR, 50ng of DNA from *H*. *pylori* was successively diluted (1 to 1:100000) in saliva from two random cases (#3 and #10) that were shown previously to be negative for the presence of *H*. *pylori*. The PCR allowed the amplification of the expected product for all different dilutions.

Additionally, and since we only detected the presence of a very-low percentage of oral positive-*H*. *pylori* from the DNA extracted from UBT positive cases, we performed a PCR experiment to validate the sensibility of our PCR results. Noteworthy, the primers that were used in this work are highly specific for *H*. *pylori*, since they recognize the *VacA* gene that is present in all strains of this bacteria.[21] To do this, we diluted 50ng of DNA extracted from a cultured *H*. *pylori* (strain 7354) in two independent saliva samples that were previously shown to be negative for *H*. *pylori*. Relevantly, this approach has proven also to be useful to decipher if the non-amplification of previous PCR in this two negative cases was caused by the possible presence of chemical inhibitors in the saliva of those two individuals. Then, we performed a successive series of dilutions in saliva of those individuals, ranging from 1:10 to 1:100000, followed by the PCR with the *VacA* primers. As shown (Fig 2), it was possible to amplify the specific *VacA*-amplicon from undiluted samples to the most diluted ones (in this case, a *H*. *pylori* DNA with a concentration of 0.5 ρg). Thus, our results indicate that the employed PCR approach to identify *H*. *pylori* proven to be highly sensitive, and the existence of possible PCR inhibitors in saliva samples, if they exist, played an insignificant role in the amplification process. Finally, we confirmed the identity of the PCR product by nucleotide sequencing.

## Discussion

When analyzing the prevalence of gastric *H*. *pylori*, the portuguese adolescents included in the present study showed a higher prevalence of infection when compared with other studies, namely by Mana *et al*.(11.0%) in Belgium and Sousa *et al*. (24.9%) in Brazil, but on the other hand registered a lower prevalence when compared with studies developed by Constanza *et al*. (47.6%) in Mexico.[22–24] The results of the present study demonstrated that the adolescents that live in urban areas and with worse socioeconomic status have a higher prevalence of gastric *H*. *pylori* infection, which may be related with poorer living conditions, lack of hygiene education and running water contamination.[5,25] The prevalence of *H*. *pylori* was also higher among older adolescents, which is also verified in the study developed by Miranda *et al*. and Kodaira *et al*.[26,27]

Our results reveal a very low prevalence of *H*. *pylori* in the oral/salivary niche, which leaves the question if the young age of the sample studied can justify such a low prevalence of *H*. *pylori* present in the oral cavity. One possible explanation may be the fact that the presence of the bacterium in the oral cavity might only happen after a certain period of time after the appearance in the stomach. The direct relation between oral/salivary *H*. *pylori* and age is not well established and must be studied in future research.

The prevalence of oral/salivary *H*. *pylori* verified in the present study was lower than the results obtained in studies developed by Fernando *et al*. (27.7%) and Wichelhaus *et al*. (82.0%).[28,29] The differences observed in the prevalence of oral *H*. *pylori* may be justified by the diverse methodologies/techniques employed in the different studies to detect the bacteria.[30] Another possible justification relay on the fact that the adolescents involved in this study do not have gastric pathology diagnosed nor they have symptoms compatible with *H*. *pylori* infection, which contrasts with some of the previous studies that use adults as study targets, and that present a clear diagnosis of gastric pathology associated with *H*. *pylori* infection.[4,10, 31–33] Also, in the present study, the detection of oral *H*. *pylori* was accomplished by the direct amplification of a portion of a specific gene of the bacteria: *VacA*. All the tests that identified the specific gene *VacA* were considered positive for the presence of *H*. *pylori* in the oral cavity. What we need to understand is that *H*. *pylori* is not the only microorganism that produces urea.[13,14] Importantly, when identifying the presence of oral *H*. *pylori* through the amplification of the urease gene by PCR there is a high risk of detecting other microorganisms, such as *Streptococcus salivarius*.[34] So, the amplification of the urease gene to identify *H*. *pylori* in the oral cavity may overvalue the true prevalence of this bacteria in that niche. Moreover, this fact can justify the differences of prevalence of *H*. *pylori* in the oral cavity when comparing the present results with those presented in other studies.[10,13, 35, 36] Therefore, the low prevalence of *H*. *pylori* in the oral cavity of the present study is justified by the highly specific methodology applied characterized by the identification of the *VacA* specific for *H*. *pylori* and identified by PCR.

Medina *et al*., referred that there may be differences in results when analyzing the presence of *H*. *pylori* in dental plaque and saliva.[37] Another reason that could underlie the infrequent detection of *H*. *pylori* on DNA samples derived from saliva can reside in low amount of that bacteria in that particular niche, which will hamper the reliable identification, as stated previously.[18,19] Nevertheless, and corroborating our results regarding clinic and pathological features, Burgers *et al*. verified that the occurrence of *H*. *pylori* in the oral cavity was not found to be correlated neither to any general or oral health parameters.[9], an observation that was also verified in our study. Kabir suggested that the detection rate in saliva was lower than that observed in feces, making saliva a less suitable specimen for the diagnosis of *H*. *pylori* infection.[38]

The results obtained in the present study demonstrate a lack of significant correlation between the presence of oral and gastric *H*. *pylori*, since only three cases of 157 participants with UBT positive test for gastric *H*. *pylori* were also positive for oral/salivary *H*. *pylori*. Therefore, at least among adolescents the oral cavity may not be considered a *reservoir* for *H*. *pylori* in individuals with gastric infection.[6,39] Interestingly, some authors suggested that *H*. *pylori* is not consistently present in the oral cavity environment, but is only transiently present due to contaminated ingested food or because of the uprising of the bacteria due to gastroesophageal reflux.[2,30]

An important issue that still needs to be well studied and determined is the influence of the putative oral *H*. *pylori* presence on dental caries development and oral health status. The results of this study also demonstrated a lack of association between *H*. *pylori* infection and the prevalence of dental caries. Namiot *et al*., referred that the occurrence of *H*. *pylori* antigens in dental plaque of natural teeth is not associated with oral health status or dental plaque removal practices from both natural teeth and removable dentures.[30] Berroteran *et al*., reported a lack of correlation between *H*. *pylori* infection and dental hygiene, dental caries, periodontal disease or use of dentures.[40] The diversity of results of various studies established the necessity of developing more studies in order to understand the role and consequences of the presence of *H*. *pylori* in the oral cavity, mainly among adolescents. A review article developed by Al-Sayed *et al*., confirmed the need of more epidemiological studies in order to determine the real quantification of *H*. *Pylori* in the oral cavity and understand its importance in human health.[41]

## Conclusions

Considering the results of the present study, we can conclude that the prevalence of oral *H*. *pylori* is very low, even among those with a positive test for gastric *H*. *pylori*, which reveals that, in this specific sample composed by adolescents, the oral cavity cannot be considered a *reservoir* for infection and re-infection of *H*. *pylori*, and cannot be considered for diagnosis of gastric *H*. *pylori*. The present study reveals that there is no significant correlation between the occurrence of *H*. *pylori* infection in the stomach and the oral health of adolescents not verifying any association with the presence of dental caries. However, the presence of the gastric *H*. *pylori* infection may be correlated with socio-demographic variables such as age, residence area and socioeconomic status. Nevertheless, it is urgent to perform more studies in samples from different age-clusters, and not only in adolescents, in order to understand the possible biological role of *H*. *pylori* in the oral cavity.

## Supporting Information

S1 File (DOC)
