# Supplementary material for: Oral and Gastric Helicobacter Pylori: Effects and Associations
Source: PLoS One. 2015 May 26;10(5):e0126923. doi: 10.1371/journal.pone.0126923 (PMC4444322; doi:10.1371/journal.pone.0126923)
Supplement: S1 File — (DOC) [file pone.0126923.s001.doc]

**Supporting Information Legends**

**Table A in S1 File: Prevalence of gastric *H. pylori* among adolescents*.* (detected=positive test; non-detected=negative test).**

|  | N | % |
| --- | --- | --- |
| **Gastric *H. Pylori*** |  |  |
| Positive | 157 | 35.9 |
| Negative | 280 | 64.1 |

**Table B in S1 File: Association between the presence of gastric *H. pylori* and dental caries.**

|  | Carie-free | | | At least 1 carie | |  |
| --- | --- | --- | --- | --- | --- | --- |
|  | N | % | N | | % | *p* |
| **Gastric *H. Pylori*** |  |  |  | |  |  |
| Positive | 92 | 62.6 | 55 | | 37.4 |  |
| Negative | 159 | 59.8 | 107 | | 40.2 | 0.3 |

**Table C in S1 File: Prevalence of salivary *H. pylori* among adolescents with positive test for gastric *H. pylori.* (detected=positive test; non-detected=negative test).**

|  | Positive for salivary *H. Pylori* | | | Negative for salivary *H. Pylori* | |
| --- | --- | --- | --- | --- | --- |
|  | N | % | N | | % |
| **Gastric *H. Pylori*** |  |  |  | |  |
| Positive | 3 | 1.9 | 154 | | 98.1 |
